# The role of plasmodesma-located proteins in tubule-guided virus transport is limited to the plasmodesmata

**DOI:** 10.1007/s00705-016-2936-2

**Published:** 2016-06-23

**Authors:** P. W. den Hollander, S. N. Kieper, J. W. Borst, J. W. M. van Lent

**Affiliations:** 1 Laboratory of Virology, Wageningen University, Droevendaalsesteeg 1, 6708 PB Wageningen, The Netherlands; 2 Laboratory of Biochemistry, Microspectroscopy Centre, Dreijenlaan 3, 6703 HA Wageningen, The Netherlands

## Abstract

Intercellular spread of plant viruses involves passage of the viral genome or virion through a plasmodesma (PD). Some viruses severely modify the PD structure, as they assemble a virion carrying tubule composed of the viral movement protein (MP) inside the PD channel. Successful modulation of the host plant to allow infection requires an intimate interaction between viral proteins and both structural and regulatory host proteins. To date, however, very few host proteins are known to promote virus spread. Plasmodesmata-located proteins (PDLPs) localised in the PD have been shown to contribute to tubule formation in cauliflower mosaic virus and grapevine fanleaf virus infections. In this study, we have investigated the role of PDLPs in intercellular transport of another tubule-forming virus, cowpea mosaic virus. The MP of this virus was found to interact with PDLPs in the PD, as was shown for other tubule-forming viruses. Expression of PDLPs and MPs in protoplasts in the absence of a PD revealed that these proteins do not co-localise at the site of tubule initiation. Furthermore, we show that tubule assembly in protoplasts does not require an interaction with PDLPs at the base of the tubule, as has been observed *in planta*. These results suggest that a physical interaction between MPs and PDLPs is not required for assembly of the movement tubule and that the beneficial role of PDLPs in virus movement is confined to the structural context of the PD.

## Introduction

Plant viruses spread from initially infected cells to neighbouring uninfected cells through cell-wall-spanning channels called plasmodesma (PD, plural plasmodesmata, PDs). Native PDs regulate the transport of macromolecules between cells and do not allow passage of virions or viral genomes [1]. Therefore, plant viruses encode specialized proteins called movement proteins (MPs), which modify the PD to allow passage of viruses or their genomes. Viruses that transport their genomes between cells as mature virions need to modify the structure of the PD pore to accommodate formation of a movement tubule [2], a process that requires the action of both the viral MP and host proteins [3, 4]. Tubule-guided virus transport is exemplified by icosahedral RNA viruses such as cowpea mosaic virus (CPMV) [5] and grapevine fanleaf virus (GFLV) [6].

Although the substitution of luminal PD components by a viral movement tubule requires severe structural PD remodelling, very little is known about the host proteins that allow or facilitate the assembly of the movement tubule inside the PD. Proteins such as remorin [7], class 1 reversibly glycosylated polypeptides [8], calreticulin [9] and plasmodesmata-located proteins (PDLPs, [10]) were all found to localise to the PD and show an interaction with viral MPs. However, only for PDLPs a positive regulatory function in viral transport has been shown, while the function of the other PD proteins negatively affects viral spread.

PDLPs were identified through proteomic screening of *Arabidopsis thaliana* cell wall proteins [11] and characterized by Thomas and co-workers [10]. They found that PDLPs exclusively localise to the PD when expressed under their native promoter. PDLPs have a typical architecture: a short C-terminal cytoplasmic domain, a transmembrane domain, and an extensive extracellular N-terminal domain. Furthermore, all eight *Arabidopsis* PDLP isoforms interact with the MPs of GFLV and cauliflower mosaic virus (CaMV) at the base of the movement tubule constructed in the PD [12]. The interaction between GFLV MP (2B) and PDLPs was shown to be required for tubule formation, as tubule formation was significantly reduced in a triple PDLP knockout line of arabidopsis [12]. Correct localisation of PDLP to the PD greatly enhanced tubule formation of GFLV, whereas inhibition of PD localisation of PDLP completely blocked 2B localisation and tubule formation at the PD [13]. It has been suggested that PDLPs might serve as a PD recognition site for 2B and facilitate the anchoring of the movement tubule in the plasma membrane lining the PD. The structural topology of PDLPs, including apoplastic and transmembrane domains as well as a cytoplasmic carboxy-terminus that directly interacts with GFLV movement tubules, supports the proposed function of these proteins in tubule anchoring inside the PD.

To test whether the interaction with PDLPs is a general feature of tubule-forming MPs, we employed Förster resonance energy transfer (FRET) detected by fluorescence lifetime imaging (FLIM) to visualize whether the MP of CPMV also interacts with PDLPs in the PD. Furthermore, we investigated whether the proposed functions of PDLP, i.e., PD recognition, initiation of MP accumulation, and tubule anchoring, are intrinsic properties of these proteins by exploring these functions in protoplasts, plant cells that do not have a cell wall or PDs. Our results show that PDLP interacts with the MP of CPMV *in planta* in a similar fashion as has been described for GFLV and CaMV. In protoplasts, however, MP accumulations did not localise with the PDLP, and no PDLP could be detected at the base of the movement tubules formed at the protoplast surface.

## Materials and methods

### Plant material

*Nicotiana benthamiana* (Nb) plants were grown on soil in a climate-controlled growth chamber at 70 % humidity under a long photoperiod regime (16 h light, 8 h dark) at temperatures of 22 °C (±1°). Wild-type and triple-PDLP-knockout (PDLP^−123^) *Arabidopsis thaliana* plants (ecotype Col-0; [12]) were grown under the same conditions at 20 °C (±1°).

### Constructs

The plasmids containing an N-terminal fusion of GFLV 2B MP to GFP (GFP-2B) and *Arabidopsis thaliana* PDLP1-GFP and PDLP1-RFP were obtained from Dr. Khalid Amari and have been described previously [12]. A fusion of GFP to the C-terminus of CPMV MP was created in the binary vector pSOL2095 [14]. The 48K reading frame from the pMON-MP-GFP vector [15] was amplified by PCR using Phusion polymerase (Thermo Scientific) and the following primers containing AttB sites (underlined) to allow subsequent gateway (Invitrogen) cloning: Fw (5’ to 3’), GGGGACAAGTTTGTACAAAAAAGCAGGCTTAACCATGGAAAGCATTATGAGCCG; Rv (5’ to 3’), GGGGACCACTTTGTACAAGAAAGCTGGGTATTGTGGAAAAGCCA-CATTC. The amplified fragment was inserted into the pDonor207 vector and the 48K-containing pDNOR207 plasmid was recombined with the pSOL2095 binary vector. The sequence of the fusion construct in the pSOL vector was verified. For visualisation of the endoplasmic reticulum (ER) a 35S promoter-driven GFP-HDEL construct was used, which expresses GFP with the -HDEL ER retention signal fused to its C-terminus [16].

### *Agrobacterium tumefaciens*-mediated transient protein expression in *N. benthamiana*

Transformed *A. tumefaciens* (LBA4404, carrying 48K-GFP, GFP-2B or PDLP1-GFP constructs, and GV3101 carrying the PDLP1-GFP construct) were used at an OD_600_ of 0.5 in an *A. tumefaciens* transient transformation assay (ATTA) performed as described previously by de Ronde and co-workers [17]. Leaves of 4- to 5-week-old *N. benthamiana* plants were infiltrated with bacterial suspensions, and fluorescent signals could usually be detected 2 days post-ATTA. Co-infiltration of bacterial suspensions containing different constructs was done by mixing the suspensions in a 1:1 ratio. Microscopic analysis of the infiltrated area was done 3 or 4 days post-ATTA.

### Isolation and transformation of protoplasts

Protoplasts were isolated from young leaves, 4 cm in length and 3.5 cm in width (± 0.5 cm), of 3- to 4-week-old *N. benthamiana* plants. These leaves were cut in a featherlike pattern of 1-mm-wide strips from the midvein outward. The leaves were then placed with their abaxial side in an enzyme solution to release mesophyll protoplasts, which subsequently were isolated as described previously [18]. Introduction of plasmid DNA into the protoplasts was done by PEG-mediated transfection with 5 µg of plasmid (per construct) per 10^5^ protoplasts [18]. Preparation of *A. thaliana* protoplasts and subsequent DNA transfection were done as follows: Arabidopsis leaves (fifth to ninth leaf) were harvested, and their abaxial epidermis was removed using the “tape-arabidopsis sandwich method” [19]. Isolation of protoplasts was done according to the protocol described by Sheen (A transient expression assay using Arabidopsis mesophyll protoplasts (http://genetics.mgh.harvard.edu/sheenweb/)), with some modifications. Leaves from which the abaxial epidermis was removed were incubated in the described enzyme solution containing adjusted amounts of enzymes (1 % [w/v] cellulase, 0.25 % [w/v] macerozym, both R10 by Serva) for 2-3 h at room temperature, while gently swirling. Protoplasts were washed three times in W5 medium prior to transfection. Per 10^5^ protoplasts, 10 µg of plasmid DNA was added and mixed for 30 s prior to the addition of 500 µl of 40 % PEG solution (PEG, MW 3,350, in 0.2 M (D)-mannitol with 100 mM Ca(NO_3_)_2_). Protoplasts, DNA and PEG were mixed for 30 s, diluted with 4.5 ml of W5, mixed by inversion, and incubated at 25 °C for 15 min. After two additional washes, protoplasts were stored in W5 medium with 50 µg of gentamicin per ml until inspection at 24 h post-transfection.

### Confocal microscopy

Infiltrated leaf sections were placed in an imaging chamber filled with perfluorodecalin (Sigma). This chamber consisted of two coverslips sealed with perfluorinated grease (RT15, Fomblin). Protoplasts were imaged by sandwiching a droplet of suspension between two coverslips spaced 0.5 mm apart. Confocal imaging of leaves and protoplasts was done using a Zeiss LSM 510-META confocal laser scanning microscope with a 63x/1.4 plan-apochromat oil immersion lens. The microscope was operated in multi-channel mode, sequentially exciting GFP (488 nm argon-laser, 5 % laser power) and RFP (543 nm helium-neon laser, 30-50 % laser power), and their emission was detected at 505-530 nm and 560-615 nm, respectively. Callose was detected by infiltration of leaf material with an aniline blue solution 0.1 % (w/v) in 67 mM K_2_HPO_4_, pH 9.0 (Merck). Co-localisation of signals was quantified by visual inspection of the presence or absence of a PDLP signal at the site of MP-GFP accumulation.

### FRET-FLIM measurements

Förster resonance energy transfer (FRET) is a photo-physical process in which the excited-state energy from a fluorescent donor molecule is transferred non-radiatively to an acceptor molecule. FRET is based on weak dipole–dipole coupling and only occurs if donor and acceptor are in very close proximity (<10 nm, [20]). There are several methods to quantify and visualize FRET. Donor fluorescence lifetime imaging (FLIM) is the most straightforward approach, since the fluorescence lifetime is a concentration-independent property. However, fluorescence lifetimes are sensitive to the environment, which is the basis for FRET-FLIM analysis. Typically, FRET-FLIM experiments consist of measuring donor fluorescence lifetimes (here GFP) in the absence (***τ***_*D*_) and presence (***τ***_*DA*_) of acceptor molecules (here RFP) resulting in spatially resolved color-coded fluorescence lifetime images. Observation of a decreased donor fluorescence lifetime is used as read-out for molecular interactions [21, 22].

Time-correlated single-photon-counting FLIM measurements were done on a Leica SP5X-SMD multi-mode confocal laser scanning microscope using a 63x water immersion 1.2NA lens. GFP/RFP were excited using a white-light laser (WLL; or super continuum laser), which emits a continuous spectrum from 470 to 670 nm, within which any individual excitation wavelength in 1-nm increments can be selected. Confocal imaging was performed using internal filter-free spectral photomultiplier tube detectors. GFP and RFP were sequentially excited using WLL GFP at 488 nm (10 % laser power) and RFP at 554 nm (8 % laser power). Fluorescence was detected at a wavelength of 505-545 nm for GFP and 560-615 nm for RFP. For FRET- FLIM experiments, the WLL (488 nm) at a pulsed frequency of 40 MHz was used. For recording of donor fluorescence, an external fibre output was connected to the Leica SP5 X scan head and coupled to a Hamamatsu HPM- 100-40 Hybrid detector (Becker & Hickl), which has a time resolution of 120 ps. Selection of GFP fluorescence was performed using a bandpass filter at 505-545 nm. Images with a frame size of 128 × 128 pixels were acquired with acquisition times of up to 90 s. From the fluorescence intensity images, the decay curves were calculated per pixel and fitted with either a single or double exponential decay model using SPCImage software (Becker & Hickl, version 3.2.3.0). The mono-exponential model function was applied for donor samples with only GFP present. For samples containing two fluorophores, GFP and RFP, a 2-exponential model function was used without fixing any parameters.

Data were analysed using the SPC image, and FRET efficiencies were calculated using the equation

$$ {\text{E}} = \left( {\frac{{R_{0}^{6} }}{{R_{0}^{6} + R}}} \right) = 1 - \frac{{\tau_{DA} }}{{\tau_{D} }} $$

where *R*_*0*_ is the Försters radius, *R* is the distance between donor and acceptor and τD and τDA are the lifetime of GFP in the absence and presence of RFP acceptor, respectively.

### Statistical analysis

To determine whether the decrease in fluorescent lifetime in the presence of an acceptor molecule was statistically significant, the non-normally distributed lifetime data were analysed by Mann–Whitney–Wilcoxon tests. These tests showed a significant (*P* < 0.001) decrease in lifetime of both 48K-GFP and GFP-2B when in the presence of PDLP-RFP acceptor molecule.

## Results

### Transiently expressed MPs localise to plasmodesma and form tubules *in planta*

**Fig. 1:**
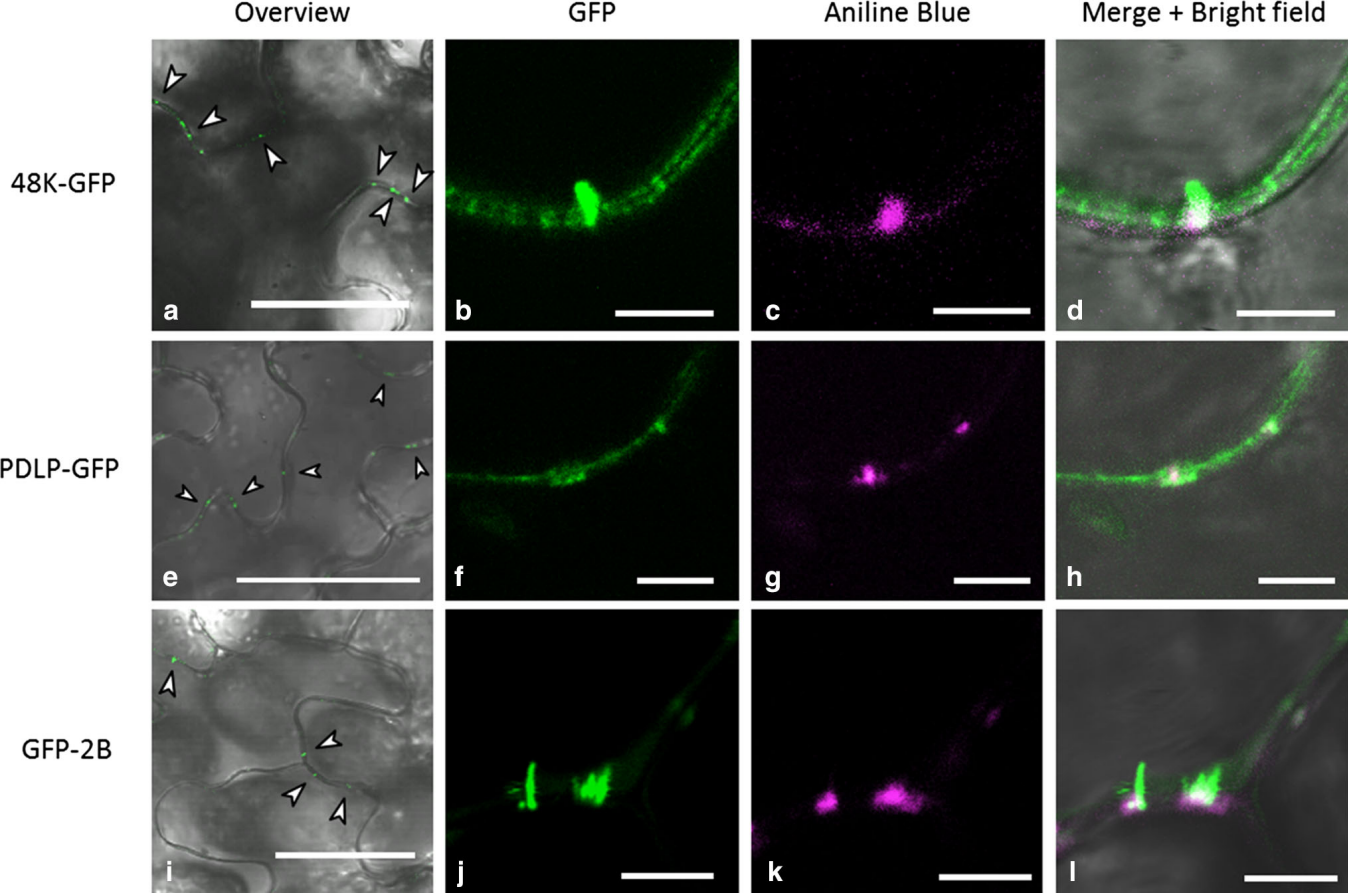
Transiently expressed PDLP and MP localise to plasmodesmata. GFP-labelled 48K, PDLP and 2B localise as punctate spots at the cell wall, indicated by arrowheads in the overview panels (a, e, i). Detailed confocal images show that movement tubules formed by 48K-GFP (b-d) and GFP-2B (j-l) localise in plasmodesmata, which are identified by callose staining using alinine blue (c, k). Also, PDLP-GFP localised to PDs, as was confirmed by callose staining (f-h). Imaging was done 3 days post-ATTA. Scale bars in a, e, and i are 50 µm; scale bars in b-d, f-h and j-l are 5 µm

To test whether CPMV MP (48K) interacts with PDLPs that are located in PDs, a C-terminal fusion of GFP to the 48K protein (48K-GFP) was constructed. Confocal microscopy of transformed epidermal cells revealed that most 48K-GFP accumulated in punctate spots at the cell wall (Fig. 1a) and, to a lesser extent, formed fluorescent tubules across the PD (Fig. 1b), which were visualized by aniline blue staining of callose (Fig. 1c and d). Apparently, the C-terminal fusion of GFP to the 48K MP does not hamper its localisation to the PD nor its assembly into tubules, indicating that this fusion protein is fully functional and suitable for *in planta* experiments. The expression of GFP fusions to PDLP (PDLP-GFP, Fig. 1e to h) and GFLV 2B MP (GFP-2B, Fig. 1i to l) also resulted in the formation of punctate spots (PDLP and 2B) and cell-wall-spanning tubules (2B).

### The movement protein of CPMV interacts with PDLP at the PD

**Fig. 2:**
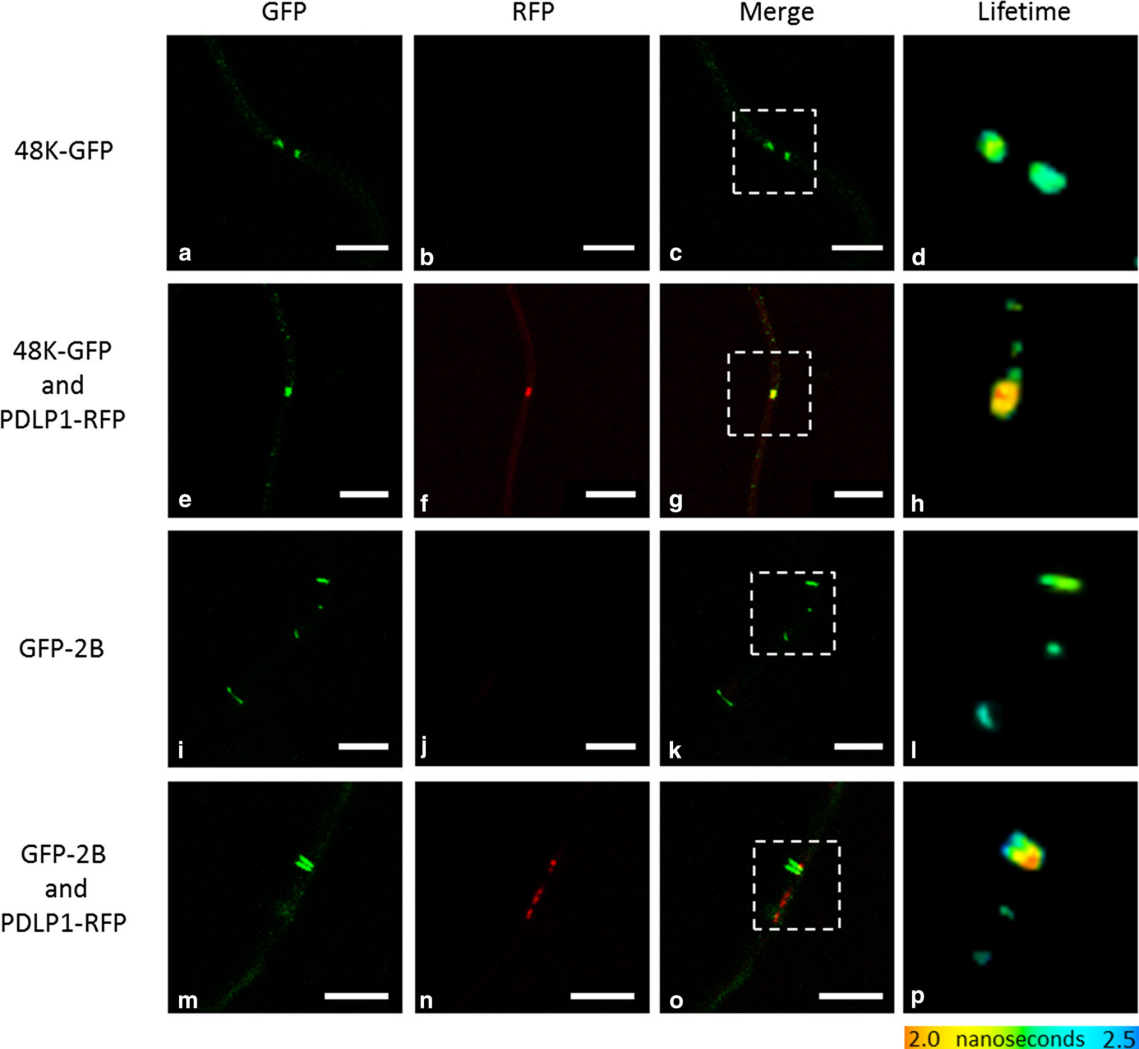
Interactions of 48K-GFP and GFP-2B with PDLP-RFP in PDs. Confocal images showing the location and fluorescence lifetime of GFP-labelled CPMV 48K MP, either in the absence (a-d) or presence of PDLP-RFP (e-h). The localisation and lifetime of GFLV 2B MP are also presented in the absence (i-l) and presence of PDLP-RFP (m-p). Reduced fluorescence lifetimes for 48K-GFP in the presence of PDLP-RFP can be seen in h (compare lifetime to d), and GFP-2B in p (compare lifetime to l). Lifetime image panels (right column) display a pseudo-coloured image representing the GFP lifetime, as indicated by the colour scale below the column. White dashed boxes indicate spots portrayed in lifetime image. Scale bar = 5 µm

To establish whether PDLPs and 48K proteins co-localise and interact with each other, a representative member of the arabidopsis PDLP family (PDLP1) was fused to RFP (PDLP-RFP) and transiently expressed along with 48K-GFP in *N. benthamiana* leaves (Fig. 2a to c and e to g). As a positive control, GFP-2B and PDLP-RFP, two proteins that are known to interact in the PD, were co-expressed (Fig. 2i to k and m to o). In transformed leaf cells, bright fluorescence of both 48K-GFP and PDLP-RFP could be observed in overlapping spots in the cell wall, showing co-localisation of these proteins in the PD (Fig. 2e to g). As expected, the expression of both PDLP-RFP and GFP-2B resulted in co-localisation at the base of movement tubules formed in the PD by 2B (Fig. 2m to o).

**Fig. 3:**
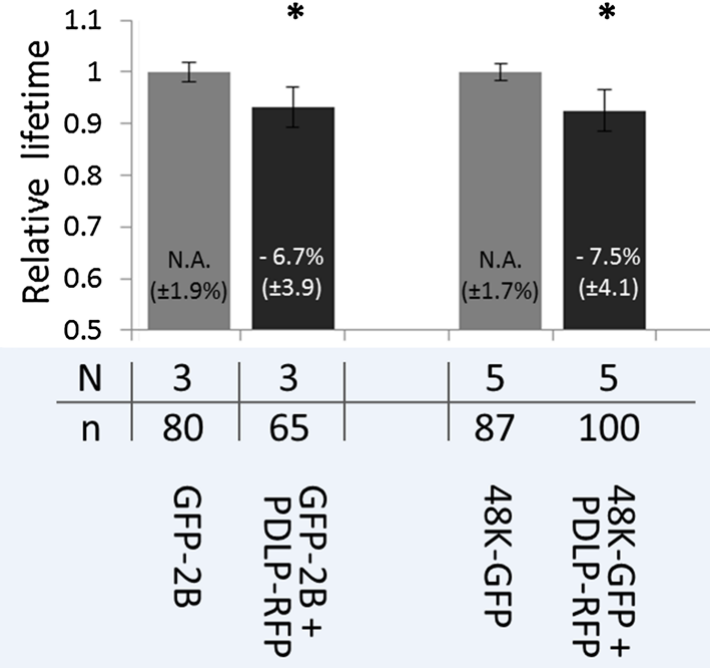
Relative fluorescence lifetime of GFP-MP fusions. Normalised fluorescence lifetime of GFP photon donor (48K-GFP and GFP-2B fusions) (grey bars) and relative fluorescence lifetime of donors in the presence of the PDLP-RFP photon acceptor (black bar). The percentage of decrease in fluorescence lifetime and standard deviations are displayed numerically in each bar. Error bars indicate the standard deviation. An asterisk indicates a significant (*P* < 0.05) decrease in the donor fluorescence lifetime. N = number of experiments, n = number of fluorescent spots measured

To establish whether co-localisation of MPs and PDLP signified a physical interaction between these proteins, FRET-FLIM experiments with MP-GFP as a donor molecule and PDLP-RFP as an acceptor molecule were conducted. As FRET only occurs if donor and acceptor molecules are in close proximity (<10 nm, [20]), the transfer of energy between the fluorophores corresponds to a molecular interaction of the fused proteins. FRET was determined by fluorescence lifetime imaging (FLIM) of the donor molecule (GFP), as the fluorescence lifetime of the donor decreases if its energy is transferred to an acceptor molecule. FRET-FLIM measurements showed that co-localisation of either 48K-GFP or GFP-2B with PDLP-RFP (Fig. 2g and Fig. 2o, respectively) coincided with a significant decrease in GFP fluorescence lifetime compared to the fluorescence lifetime of individually expressed 48K-GFP and GFP-2B proteins (compare Fig. 2d and h and Fig. 2i and p, respectively), which implies an interaction between PDLP and these MPs. The decrease in donor fluorescence lifetimes for both MPs in the presence of PDLP-RFP is summarised in Figure 3.

### Movement proteins do not co-localise with PDLP in protoplasts

**Fig. 4:**
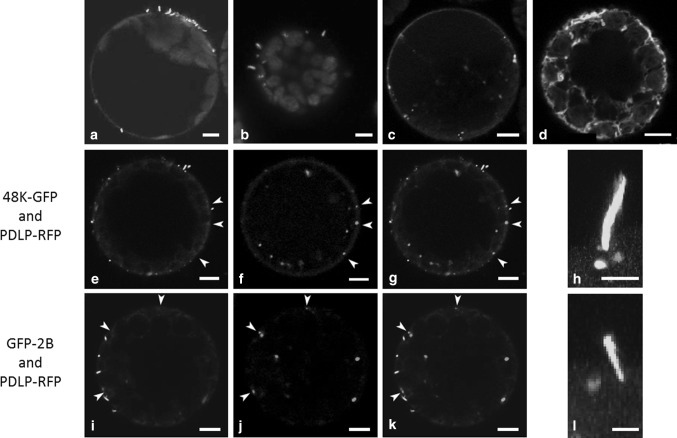
Localisation of PDLP and MPs in *N. benthamiana* protoplasts. a) 48K-GFP expressed from pSOL2095 localises to peripheral spots and short tubules. b) GFP-2B forms protruding tubules when transiently expressed in protoplasts. c) PDLP-GFP localisation to peripheral spots and, to a lesser extent, in internal spots. d) Localisation of internal PDLP-RFP spots with the ER. The latter is visualized using GFP-HDEL. e to g and i to k) Localisation of co-expressed GFP-labelled MPs (e, i) and PDLP-RFP (f, j) (merge in g and k). Arrowheads indicate peripheral PDLP spots. h and l) Extended focus image showing fluorescent 48K and 2B movement tubules (h and l, respectively) and PDLP-RFP at distinct locations, without overlap at the base of the tubule. Chloroplast autofluorescence (a, b) is displayed in magenta. The scale bar in a-g and i-k is 5 µm. The scale bar in h and l is 2 µm

To further investigate the interaction between PDLP and MPs, fluorescent protein fusions were expressed in *N. benthamiana* protoplasts. Protoplasts are isolated plant cells that are devoid of a cell wall, and consequently, PDs are absent. This allows investigation of intrinsic properties of PDLP that are independent of the structural context of the PD. Transient expression of 48K or 2B MPs resulted in outgrowth of movement tubules from the protoplast surface (Fig. 4a and b). Co-expression of PDLP and MP in protoplasts would reveal whether PDLPs direct the accumulation of MP at the plasma membrane and whether anchoring of the movement tubule base to the plasma membrane requires PDLP.

In protoplasts, PDLP-GFP formed both peripheral and internal punctate spots (Fig. 4c and d). Expression of the 48K-GFP from either pSOL (Fig. 4a) or pMON (Fig. 4e to h) vector or GFP-2B (Fig. 4b and Fig. 4i to l) resulted in formation of peripheral punctate spots and fluorescent tubules protruding from the protoplast surface. Thus, all three proteins: 48K-GFP, GFP-2B and PDLP-RFP, localised to peripheral spots. However, co-expression of PDLP-RFP with either 48K-GFP or GFP-2B did not result in co-localisation (Fig. 4e to g and Fig. 4I to k). Close inspection of the assembled tubules showed that PDLP-RFP was not found at the base of either 48K or 2B tubules (Fig. 4h and Fig. 4l, respectively). Quantification of the co-localisation between PDLP and MPs leads to the conclusion that both 48K-GFP and GFP-2B show high co-localisation fractions with PDLP-RFP in plant cells; however, co-localisation of the two respective proteins in protoplasts was only occasionally observed (Table 1). The large number of movement tubules formed on protoplasts despite the rare occurrence of co-localisation suggests that a PDLP interaction is not essential for tubule formation in protoplasts.

**Table 1:** Quantification of co-localisation of MPs with PDLPs in plants and protoplasts. Levels of co-localisation observed between the CPMV (48K) and GFLV (2B) MPs and PDLP-RFP in the plasmodesmata of plant cells and in protoplasts are shown. Co-localisation levels represent averaged values from three pooled experiments, obtained by quantification of at least 15 cells in each experiment

	Co-localisation (in %)	Standard deviation (in %)
**In plasmodesmata**		
48K-GFP with PDLP-RFP	98.4	±3.6
GFP-2B with PDLP-RFP	97.2	±5.1
**In protoplasts**		
48K-GFP with PDLP-RFP	5.4	±7.3
GFP-2B with PDLP-RFP	4.9	±7.4

**Fig. 5:**
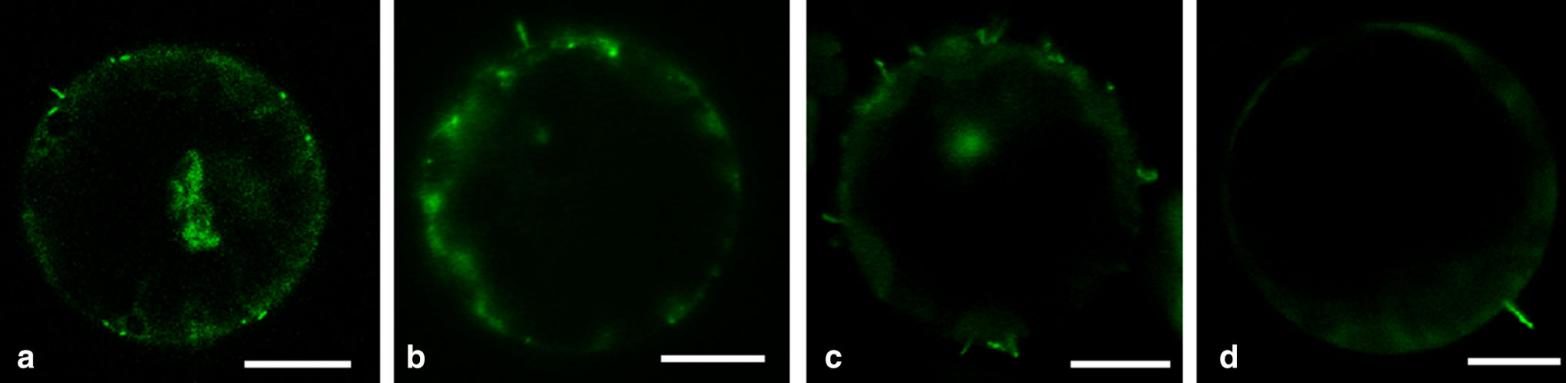
Tubule formation of 48K-GFP and GFP-2B in arabidopsis protoplasts. a and b) Wild-type *A. thaliana* protoplasts transiently expressing 48K-GFP (a) or GFP-2B (b) show the outgrowth of fluorescent movement tubules. c and d) Triple PDLP knockout (PDLP^−123^) *A. thaliana* protoplasts expressing 48K-GFP (c) or GFP-2B (d) proteins also showing the outgrowth of viral movement tubules. Scale bar = 10 µm

Amari and co-workers [12] showed that tubule formation of GFLV was severely reduced in a triple PDLP knockout genotype of *Arabidopsis thaliana* (At PDLP^−123^). As arabidopsis is not a host for CPMV, tubule formation of 48K-GFP could not be tested *in planta*. However, CPMV is able to infect arabidopsis protoplasts; therefore, protoplasts from the wild type and from the PDLP^−123^ genotype were transfected with 48K-GFP or GFP-2B constructs. In transfected protoplasts from both the PDLP-knockout and wild-type genotypes, tubule formation was observed (Fig. 5). Transfection with either 48K-GFP or GFP-2B constructs yielded similar levels of tubule formation in wild-type and PDLP^−123^ cells, but due to limited transfection efficiency, quantification was not possible.

## Discussion

PDLP isoforms are exclusively found in PDs. In this structure, PDLPs associate with MPs and benefit the assembly of viral movement tubules [12]. Our data show that PDLP1 co-localises and interacts with the MPs of both CPMV and GFLV at PDs in *N. benthamiana* (Fig. 2). When co-expressed in protoplasts from the same host, however, no co-localisation of, and hence no interaction between, the MPs and PDLPs was observed (Fig. 4). In both cases, the expressed MPs were competent to form movement tubules, in PDs and at the cell surface of protoplasts. The formation of peripheral punctate spots and tubules in protoplasts and the absence of co-localisation of MPs and PDLPs suggest that PDLPs are not directly involved in the accumulation of MPs or anchoring of the tubule at in the plasma membrane. These findings also suggest that the interaction between PDLP1 and the MP requires the structural context of the PD.

Transient expression of a fluorescent fusion protein consisting of the CPMV MP and GFP in *N. benthamiana* leaves showed the expected localisation of the MP as peripheral punctate spots at the cellular periphery and, to a lesser extent, in movement tubules (Fig. 1; [23]). The punctate spots may represent short tubules inserted in the PD, as the spots are retained in the cell wall upon plasmolysis (data not shown) and the 48K-GFP construct effectively forms tubules on protoplasts (Fig. 4). Thus, the expressed CPMV MP is fully competent in the formation of tubules, even though the CPMV tubules formed *in planta* are not as obvious as those formed upon expression of GFLV 2B.

Co-localisation experiments in which GFP-labelled MPs were expressed with PDLP-RFP *in planta* revealed that CPMV 48K and GFLV 2B specifically localised with PDLP-RFP in the PD. FRET-FLIM analysis showed a significant reduction in 48K-GFP fluorescence lifetime, indicating that 48K interacts with PDLP at the PD (Fig. 2). Interaction between 2B and PDLP was also observed in the PD, which is in line with previous reports [12, 13]. The FRET-efficiencies of the MP-PDLP interactions were highly similar for both 48K and 2B (7.5 % and 6.7 % respectively, Fig. 3), which suggests that the association of these movement proteins with PDLP occurs in a similar fashion. Whether this interaction is required for CPMV MP tubule formation in PD, as has been shown for the MP of GFLV, remains to be established. Expression of 48K and 2B in protoplasts of PDLP triple knockout arabidopsis plants resulted in tubule formation with an abundance and time frame similar to that observed in wild-type protoplasts (Fig. 5). Although our limited dataset does not allow detailed quantitative analysis, the presence of movement tubules in the first place implies that knockout of three PDLPs does not severely hamper tubule formation, if at all, by either 48K or 2B MP in protoplasts. The pertinent experiments require repetitions in protoplasts and tissues of PDLP-silenced plants that are permissive for CPMV infection to obtain robust data on the influence of PDLP on the tubule-forming capacity of 48K in protoplast and in PDs. However, no PDLP knockdown lines of any CPMV host plants are currently available.

The interaction of 48K with PDLPs supports the hypothesis that the interaction between MPs and PDLPs is conserved among tubule-forming viruses [12]. Testing whether MPs of viruses such as tomato spotted wilt virus (family *Bunyaviridae*) or alfalfa mosaic virus (family *Bromoviridae*), which form structurally distinct tubules [24, 25], also interact with PDLP in the PD would be very interesting, as this would support the suggested evolutionary relationships between tubule-forming viruses [26].

Although it is clear that PDLPs interact with MPs of GFLV, CPMV and CaMV in the PD (this work; [12]), the significance and underlying mechanisms of this interaction remain unclear. Because the presence of PDLP in PDs was found to be required for localisation of GFLV 2B to the PD and to enable its tubule formation, Amari *et al.* [13] suggested that PDLP family members facilitate the accumulation of MPs at PDs and anchoring of movement tubules to the plasma membrane. We have tested this hypothesis by co-localisation studies of PDLPs and viral MPs in protoplasts. As PDs are absent from such cells, we could investigate whether PDLPs serve as recognition and/or anchoring signals for MPs when not associated with the PD. PDLP and GFLV MP showed an interaction *in planta* even after the movement tubule was formed, so we assumed that this continued interaction would result in substantial co-localisation of these proteins in protoplasts. However, in protoplasts, no obvious co-localisation was observed between PDLP and either of the MPs (Table 1). We cannot completely rule out an effect of competition between putative *N. benthamiana*, PDLP-like proteins and the transiently expressed *A. thaliana* PDLP1. However, in a recent proteomic analysis of the plasma membrane-tubule complex of CPMV in *N. benthamiana* no PDLP-like proteins were identified to be part of this complex isolated from infected protoplasts [27]. This and our present results suggest that PDLPs do not serve as a recognition/retention signal for MP accumulation. Because tubules originate from peripheral spots [28], we therefore propose that PDLPs do not serve as a catalyst for tubule initiation in protoplasts, as this function would require co-localisation of these proteins, which is not the case.

A possible explanation for the lack of PDLP-directed accumulation of MPs in protoplasts, in contrast to the situation *in planta*, could be the absence of a PD-specific complex composed of various PD proteins including PDLPs. The recognition of such a complex by MPs could depend on an interaction with one of the other (currently unknown) proteins or could depend on the structural organization of this multi-protein complex inside the PD. The absence of PDLP at the base of the 48K and 2B movement tubules formed on *N. benthamiana* protoplasts, indicates that membrane anchoring of the movement tubule, which is required for directed tubule outgrowth, is not mediated by an interaction with PDLP. It is likely that another conserved protein is required for the anchoring of the movement tubule to the plasma membrane, as expression of 48K in insect cells results in tubule formation [29] and no proteins with the DUF26 domain, characteristic for PDLPs, were found in insect protein databases (protein-BLAST, NCBI.com).

The exact function of PDLP in virus movement remains to be established; however, our studies have shown that PDLP1 interacts with the MP of CPMV in a manner similar to the previously established interaction with GFLV and possibly CaMV [12]. In addition, we have shown that in protoplasts PDLP are not required for localisation or accumulation of MPs prior to tubule outgrowth and that the plasma membrane anchoring of movement tubules is not mediated by PDLP. These new insights emphasize the importance of the structural environment of the PD in the analysis of the host protein involvement in plant virus intercellular movement.
